# Long-Term Effects of Rheumatoid Arthritis Treatments on Bone Mineral Density: 8-Year-Follow-Up Data from Real-World Practice

**DOI:** 10.3390/jcm15072594

**Published:** 2026-03-28

**Authors:** Louis-Edmond Barbaro, Lindsay Bustamente, Léa Evenor, Angelina Villain, Abdellahi Vall, Roxane Fabre, Laurent Bailly, Véronique Breuil, Christian Pradier, Christian Roux

**Affiliations:** 1 Department of Rheumatology, University Côte d’Azur, 06000 Nice, France; 2 Department of Public Health, University Côte d’Azur, 06000 Nice, France; 3 Cognition Behavior Technology Laboratory, University Côte d’Azur, 06000 Nice, France; 4 LAMHESS Laboratory, Rheumatology Department, University Côte d’Azur, 06000 Nice, France

**Keywords:** rheumatoid arthritis, biological therapies, synthetic DMARD, bone mineral density

## Abstract

**Objectives**: The long-term effects of rheumatoid arthritis (RA) therapies on bone mineral density (BMD) remain incompletely characterized. We aimed to evaluate BMD trajectories over an 8-year follow-up in patients with RA treated with conventional synthetic disease-modifying antirheumatic drugs (csDMARDs) or biological DMARDs (bDMARDs) in real-world practice. **Methods**: Patients were selected from an observational RA cohort established at Nice University Hospital between 2001 and 2016. Participants were classified into two groups according to treatment regimen (csDMARD only or any bDMARD exposure). BMD was assessed by dual-energy X-ray absorptiometry at baseline and after 1, 2, 3, 5, and 8 years at the lumbar spine, femoral neck, and total hip. Longitudinal changes in BMD were analyzed using multivariable linear mixed-effects models adjusted for age, sex, body mass index (BMI), disease duration, seropositivity, glucocorticoid use, anti-osteoporosis treatment, and clinical response. **Results**: A total of 312 patients were included, of whom 181 received bDMARDs and 131 were treated exclusively with csDMARDs. BMD showed limited change during the first two years in both groups. Beyond two years, modest declines were observed at hip sites at subsequent time points, whereas lumbar spine BMD did not demonstrate significant longitudinal change in pointwise analyses. In mixed-effects models, the treatment group–time interaction was significant for lumbar spine (*p* = 0.004) and total hip (*p* = 0.04), but not for the femoral neck (*p* = 0.34), indicating differential BMD trajectories over time between treatment groups. In the csDMARD group, lumbar spine and total hip BMD decreased by a mean of 0.0006 and 0.0005 g/cm^2^ per month, respectively, whereas no significant slopes were observed in the bDMARD group. Older age was associated with lower BMD, while male sex and higher BMI were associated with higher BMD across sites. **Conclusions**: In this long-term real-world cohort, BMD remained relatively stable during the first two years of follow-up. Longitudinal analyses suggested a less pronounced decline in lumbar spine and total hip BMD trajectories among bDMARD-treated patients compared with those receiving csDMARD alone, underscoring the need for ongoing bone health monitoring in RA.

## 1. Introduction

Osteoporosis is a well-known extra-articular manifestation in rheumatoid arthritis (RA) and may decrease quality of life because of increased fracture risk [1,2]. Osteoporosis occurs mainly during early-stage rheumatism and is related to disease and inflammatory activities [3]. In patients with RA, osteoporosis is multifactorial in origin, involving steroid exposure, lack of mobility, and systemic inflammation [4].

In their early stages of RA, both localized and generalized bone loss share common inflammation-driven pathways resulting from imbalances in bone formation and resorption [5]. Osteoclasts play a central role in this process, which is mainly mediated through the receptor activator of nuclear factor-kappa B ligand (RANKL) [6]. RANKL expression is up-regulated by several local and systemic pro-inflammatory cytokines implicated in RA pathogenesis, including tumor necrosis factor α (TNFα), interleukin 1 (IL-1), and interleukin 6 (IL-6). Moreover, it involves immune cells such as T and B lymphocytes [7,8].

Over the past decade, different biological therapies used in RA have efficiently controlled disease activity and prevented bone erosion [9,10,11,12,13,14,15]. However, their impact on generalized bone loss is still unclear. Of published studies showing reduced rates of generalized bone loss in RA using biological therapies, only a limited number are long-term studies [16,17]. Most were short-term, included small sample sizes, and used heterogeneous methodologies [18,19,20,21]. Due to the scarcity of studies with a duration exceeding 1 year, there is little or no information on the long-term impact of those therapies [22,23]. In addition, differences in demographic characteristics between patients included in pivotal randomized controlled trials and those in real-world settings have been highlighted by a recent Italian study [24]. These observations further underscore the need for real-world data from observational cohorts regarding RA therapies.

Furthermore, prior investigations have predominantly focused on short-term effects of TNFα inhibitors, particularly infliximab, while data on other biological therapies remain scarce.

Therefore, we aimed to evaluate the long-term effects of RA therapies on BMD over 8 years of follow-up in real-world practice.

## 2. Materials and Methods

### 2.1. Patients

Patients were selected from an observational cohort of individuals with RA established at Nice University Hospital between 2001 and 2016. Clinical, biological, BMD measurements and radiological follow-ups were part of the regular patients’ care.

Eligible participants were required to be at least 18 years old, receive treatment with a conventional synthetic disease-modifying antirheumatic drug (csDMARD) or have an indication of biological treatment (bDMARDs), fulfill the 2010 ACR/EULAR criteria, and have a dual-energy X-ray absorptiometry (DXA) scan for hip and lumbar spine (anteroposterior view of L2-L4/Hologic QDR 4500A; Hologic, Inc., Marlborough, MA, USA; Windows XP version 12.4, reference curves: NAHNES for hip and ISOS-OFELY-GENSET for spine) performed at baseline (in the rheumatologic department of Nice) prior to treatment initiation and at least once during the follow-up [25]. Only patients assessed using Hologic equipment were included. Patients who had started biological treatment prior to the baseline DXA evaluation were excluded.

Demographic data, clinical characteristics, and treatment information were collected at each visit.

Information on anti-osteoporosis treatments (including bisphosphonates, denosumab, teriparatide, and calcium/vitamin D supplementation) was systematically collected from medical records at each visit as part of routine clinical care. These treatments were prescribed according to individual fracture risk assessment and were included as a covariate in the multivariable mixed-effects models.

Patients were categorized into two groups according to treatment exposure: those receiving any bDMARD (anakinra, tocilizumab, abatacept, rituximab, infliximab, adalimumab, etanercept, certolizumab pegol, or golimumab) and those treated exclusively with csDMARDs (methotrexate, sulfasalazine, leflunomide, or hydroxychloroquine).

Early menopause was defined as menopause occurring before the age of 40 years. Erosive disease was defined by the radiographic presence of bone erosion in the hands, wrists, and/or feet.

In this study, the index date was defined as the date of the first DXA scan; this date preceded or, in some cases, coincided with the initiation of the treatment.

Patients who had received biological therapy before the baseline DXA were excluded to ensure that biological exposure occurred after cohort entry. Patients were then classified according to exposure during follow-up (any exposure to bDMARDs versus csDMARD-only treatment). Patients who switched between different biological agents during follow-up remained in the bDMARD exposure group.

The study was conducted according to the guidelines of the Declaration of Helsinki, and approved by the French National Commission on Informatics and Liberties (ND260). All participants provided their written consent, and the study was registered at ClinicalTrials.gov (NCT03076866).

### 2.2. BMD Measurements

BMD for hip and lumbar spine was assessed by DXA scan at baseline and at follow-up visits conducted after 1, 2, 3, 5, and 8 years. Standardized measurements were obtained at the lumbar spine (vertebrae L1–L4) and hip (including the femoral neck and total hip). BMD values were expressed in g/cm^2^, and reported as T-scores and Z-scores.

Osteopenia was defined as a T-score between −1 and −2.5 standard deviations (SD) and osteoporosis as a T-score below −2.5 SD, in accordance with World Health Organization guidelines. Z-score was used in non-menopausal women.

### 2.3. Statistical Analysis

Statistical tests were performed using SAS enterprise Guide 5.1 (SAS Institute, Chicago, IL, USA). Quantitative data are described as means and standard deviation (SD), and the qualitative variables are presented as frequencies and percentages. Missing data were not replaced. Data for both groups were compared using Student’s *t*-test for quantitative variables and chi-squared tests for multi-modality variables.

The second time, we selected only BMD values at 1, 2, 3, 5, and 8 years. BMD values were compared between baseline and other time points using Student’s paired t-test within each group. A *p*-value ≤ 0.05 was considered statistically significant.

To account for all BMD measurements per patient over the follow-up period, we used a linear mixed model. The model evaluated the effects of baseline age, sex, body mass index (BMI), disease duration, time elapsed between initial and final BMD measurements, radiographic joint damage, Rheumatoid Factor or anti-citrullinated protein antibody (ACPA) positive, treatment response, anti-osteoporosis therapy, and prednisone use on BMD. Time was treated as a quantitative variable. All variables significantly associated with BMD in univariate mixed models with *p* ≤ 0.20 were included in multivariate mixed models. A backward stepwise elimination approach was applied, and variables with *p* < 0.05 were retained in the final model. The group variable, time, disease duration, response to treatment, glucocorticoid exposure, and ACPA presence, were retained in the final model regardless of their *p*-values in the univariate analysis. Subsequently, the group-time interaction was included in the model to determine the possible effect of treatment on changes in BMD. Results are presented with 95% confidence intervals.

Each analysis was repeated for the three sites of BMD measurements, i.e., lumbar spine, femoral neck, and total hip.

## 3. Results

### 3.1. Patient Characteristics at Baseline

**Figure 1:**
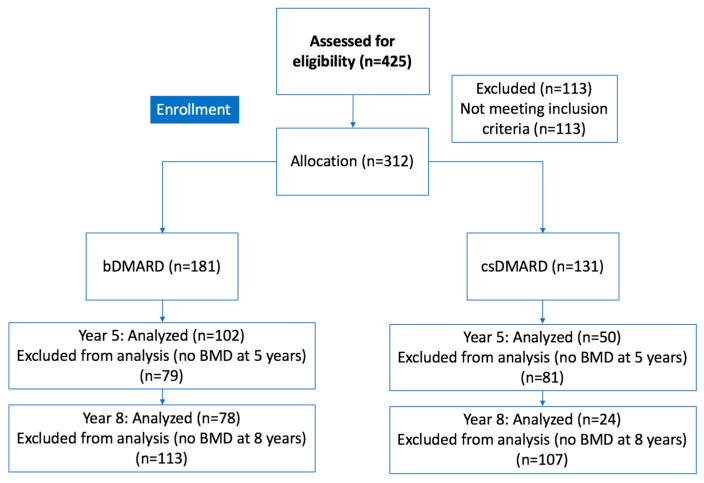
Flowchart. bDMARD: biological disease-modifying antirheumatic drug; BMD: bone mineral density; csDMARD: conventional synthetic disease-modifying antirheumatic drug.

A total of 425 patients with RA were initially screened. Of these, 113 (26.6%) were excluded due to missing baseline or follow-up BMD measurements (Figure 1).

Overall, 312 patients with active RA met the inclusion criteria. Of these, 181 patients received a bDMARD (85.1% female, mean age 58 ± 13 years old) and 131 were treated exclusively with a csDMARD (86.3% female, mean age 64 ± 12 years old). Comparison between included and excluded patients regarding sex, age, disease activity, and T-score revealed no significant differences.

Baseline characteristics, including demographic variables, clinical and therapeutic characteristics of the 312 included patients, are shown in Table 1.

During follow-up, among patients receiving bDMARDs, 50.8% were treated with a single biological therapy only, 23.8% with two agents, and 25.5% with three or more. The average duration of these treatments was 55 ± 41 months.

### 3.2. BMD Measurements

#### 3.2.1. Bone Characteristics at Baseline

At baseline, 31.5% of patients receiving bDMARDs and 29.0% of patients treated with csDMARDs had osteoporosis. Additionally, 11.6% of bDMARD-treated patients and 12.2% of csDMARD-treated patients had a history of fractures. No significant differences were observed between the two groups for these baseline parameters (*p* = 0.09 for osteoporosis and 0.87 for fracture history; Table 2 and Table 3).

#### 3.2.2. BMD Changes During Follow-Up in Comparison with Baseline

**Figure 2:**
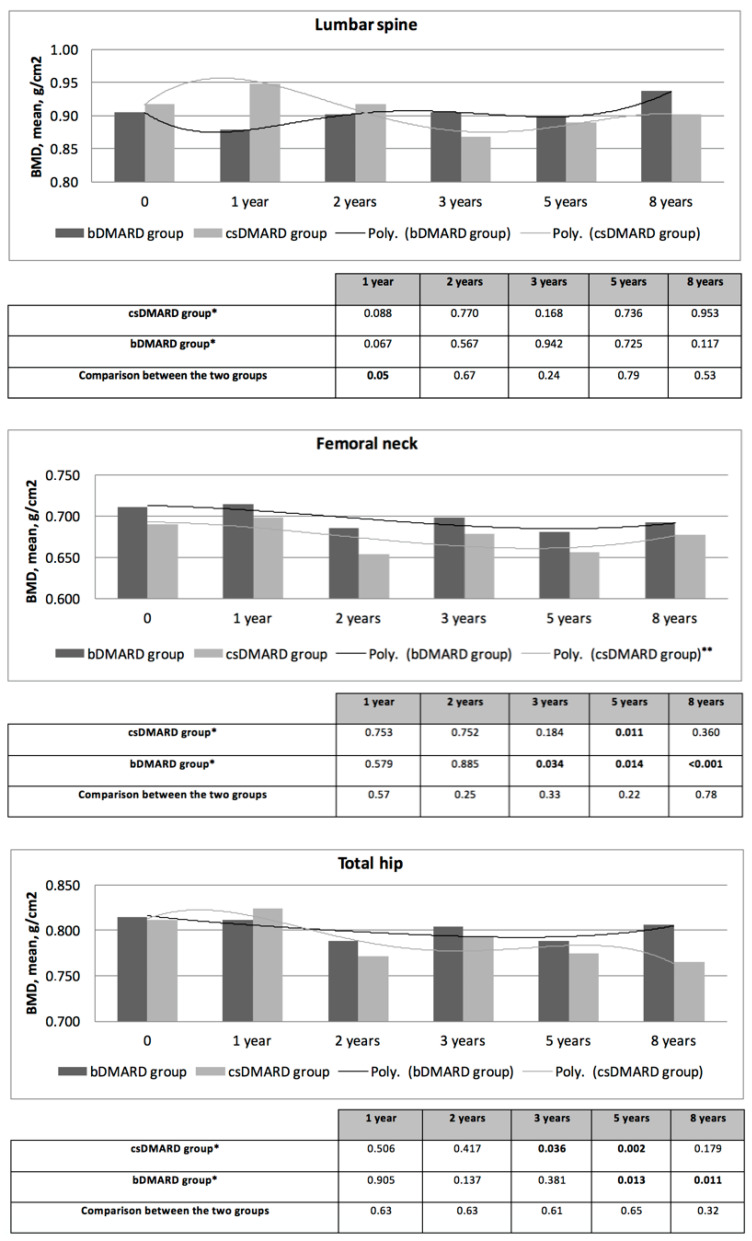
Bone mineral density (BMD) evolution at lumbar spine, femoral neck and total hip levels over a 1, 2, 3, 5 and 8-years follow-up in the biological and conventional synthetic DMARD groups and comparison between the two groups at different time of follow-up. bDMARDs: biological disease-modifying antirheumatic drug; BMD: bone mineral density; csDMARD: conventional synthetic disease-modifying antirheumatic drug; Poly.: polynomial trendline. * *p*-value of Student’s paired t-test between baseline and all other measures. ** The order of the polynomial was determined to obtain the best fit of the line to the data, as shown by R-squared value.

BMD was assessed at 1, 2, 3, 5, and 8 years and compared with baseline values (Figure 2). No significant changes were observed at 1 year in either treatment group.

At 2 years, BMD remained stable at all sites in both the bDMARD and csDMARD groups, with no statistically significant differences compared with baseline.

Beyond 2 years, modest declines in femoral neck and total hip BMD were observed at later follow-up time points in both groups, reaching statistical significance at some visits, whereas lumbar spine BMD did not demonstrate significant longitudinal change.

Between-group comparisons at each follow-up time point showed no significant differences in BMD trajectories, except at the lumbar spine at the 1-year follow-up (Figure 2).

#### 3.2.3. Factors Associated with BMD Change During Follow-Up

Multivariate linear mixed-effects models identified several factors independently associated with BMD changes over the 8-year follow-up period (Table 4).

A significant interaction between treatment group and time was observed for lumbar spine BMD (*p* = 0.004) and total hip BMD (*p* = 0.04), indicating that BMD trajectories differed over time between the csDMARD and bDMARD groups at these sites. No significant group–time interaction was found for femoral neck BMD (*p* = 0.34).

Across all skeletal sites, older age was associated with lower BMD, whereas male sex and higher BMI were associated with higher BMD values. Anti-osteoporosis treatment was significantly associated with BMD changes at all sites. Disease duration and fracture events were also independently associated with hip BMD.

Treatment response (DAS28 < 3.2) was marginally associated with lumbar spine BMD, whereas corticosteroid use was not significantly associated with BMD changes in the final models.

### 3.3. Fractures

During follow-up, 26 patients in the bDMARD group and 21 in the csDMARD group had clinical fractures. Of these, 13 bDMARD-treated patients and 14 csDMARD-treated patients had vertebral fractures, whereas 14 patients in the bDMARD group and 12 from the csDMARD group had non-vertebral fractures. No significant difference was observed between the two groups (*p* = 0.69; Table 3).

### 3.4. Effects of Different Biological Therapies on BMD

**Figure 3:**
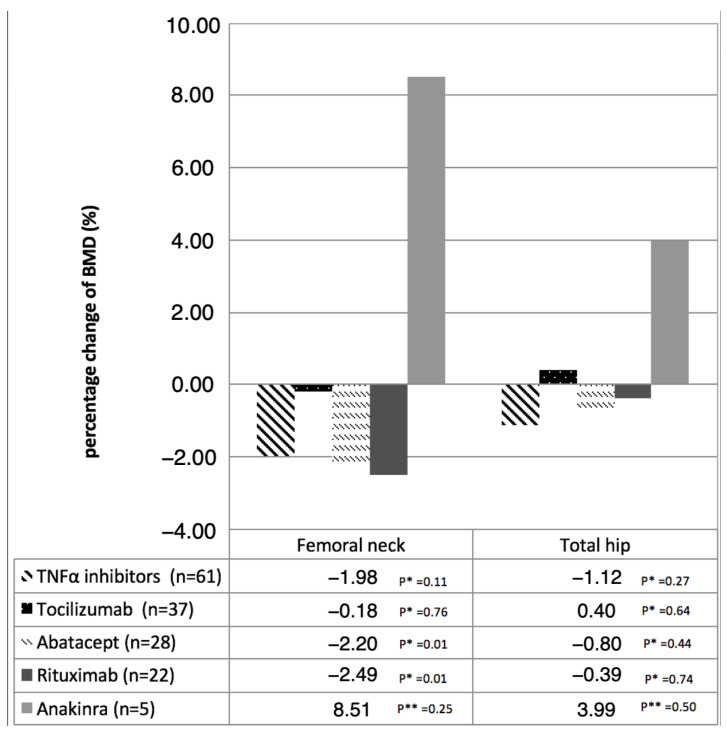
Evolution of BMD change in the femoral neck and total hip in each biological therapy group, depending on exposure time. * Intragroup comparison between baseline and final BMD—Student’s paired *t*-test; ** Intragroup comparison between baseline and final BMD—Wilcoxon signed-rank test.

In this study, only first-line biological therapies were considered. A significant BMD change was found only in patients treated with abatacept (ΔBMD = −0.016, *p* = 0.012) and rituximab (ΔBMD = −0.018, *p* = 0.013) in the femoral neck. No statistically significant changes in BMD were detected over time for patients receiving other biologic agents (Figure 3).

## 4. Discussion

Current understanding of the effects of different rheumatoid arthritis therapies on bone mineral density changes remains limited and controversial [26]. Our study contributes to this field by providing long-term real-world data on BMD trajectories over an eight-year period in RA patients treated with csDMARDs or bDMARDs.

In our study, a significant age difference was observed between the two treatment groups, with patients receiving csDMARDs being older than those treated with bDMARDs. This difference likely reflects treatment allocation in routine clinical practice rather than a selection bias inherent to the study design. Biological therapies are often prescribed more cautiously in older patients because of the higher prevalence of comorbidities and the potential risk of adverse events, particularly infections. Consequently, clinicians may be more likely to maintain conventional therapies in older individuals, whereas younger patients with more active or refractory disease are more frequently escalated to bDMARD therapy. Indeed, Vela et al. demonstrated that the incidence of the first adverse event among rheumatic patients receiving biologic therapies increased with advancing age [27]. Importantly, age was included as a covariate in the multivariable mixed-effects models in order to account for its potential influence on BMD trajectories.

During the first two years of follow-up, both treatment groups showed minimal changes in BMD at all skeletal sites, suggesting relative stabilization of bone loss during the early phase of treatment. This observation is consistent with previous studies reporting minimal short-term changes in generalized bone loss under effective disease control [18,19,20,21].

Beyond two years, modest declines in femoral neck and total hip BMD were observed at later follow-up visits in both groups. Importantly, cross-sectional comparisons at individual time points did not reveal systematic differences between treatment groups. However, longitudinal mixed-effects models indicated that BMD trajectories over time differed between csDMARD and bDMARD groups, particularly at the lumbar spine and total hip, as reflected by significant group–time interactions. Overall, these findings suggest a more favorable long-term BMD evolution in patients receiving bDMARDs compared with csDMARDs, especially after the second year of follow-up.

These differences may be related to the ability of biological therapies to more effectively suppress systemic inflammation through targeted inhibition of key cytokines involved in osteoclast activation and bone remodeling, thereby limiting inflammation-driven bone loss [28,29]. Nevertheless, modest decreases at hip sites may still occur despite biologic therapy, highlighting the multifactorial nature of osteoporosis risk in RA.

Few studies have directly compared long-term effects of csDMARDs and bDMARDs on generalized bone loss. Previous reports have suggested similar short-term effects across therapeutic strategies, whereas longer-term follow-up studies remain scarce [30,31,32]. Our findings extend these observations by demonstrating that differences in BMD evolution may emerge over time, particularly when assessed through trajectory-based longitudinal analyses.

However, these findings should be interpreted with caution. Comparisons at individual follow-up time points were largely non-significant, and the observed differences mainly emerged from trajectory-based longitudinal analyses. In addition, subgroup analyses evaluating individual biologic agents were limited by sample size and reduced statistical power, particularly at longer follow-up durations. These results should therefore be considered exploratory and require confirmation in larger prospective studies specifically designed to assess differential effects of biologic therapies on bone mineral density.

Regarding individual biologic agents, no consistent differences were observed across therapies in this cohort. The significant changes detected in small subgroups (abatacept and rituximab) should be interpreted cautiously. Indeed, due to limited sample sizes and treatment switching during follow-up, these findings cannot be interpreted with certainty and may underestimate the true effect. They should therefore be regarded as exploratory data, underscoring the need for studies specifically designed to evaluate these molecules.

The impact of bDMARDs on fracture risk also remains uncertain. Consistent with recent meta-analyses [33], we did not observe a significant difference in clinical fracture occurrence between treatment groups. Vertebral fractures may have been underestimated due to their frequently asymptomatic presentation.

From a clinical perspective, these findings suggest that effective control of systemic inflammation may contribute to limiting generalized bone loss in patients with rheumatoid arthritis. However, the modest decline observed at hip sites during long-term follow-up highlights the need for continued bone health monitoring and appropriate osteoporosis prevention strategies, regardless of the therapeutic regimen used.

This study has several limitations. Its observational design raises the potential for confounding by indication and channeling bias, as patients receiving bDMARDs had higher baseline disease activity. Although multivariable mixed-effects models were applied to adjust for relevant clinical covariates, residual confounding cannot be excluded. In addition, the association between anti-osteoporosis therapy and lower BMD likely reflects confounding by indication, as patients at higher fracture risk were more likely to receive such treatments. Missing data at later follow-up visits and potential artifacts affecting lumbar spine measurements (e.g., osteophytes, calcifications) should also be considered when interpreting the results [34,35].

Overall, our findings underscore the importance of long-term monitoring of bone health in RA patients, regardless of treatment regimen, particularly beyond the first years of therapy.

## 5. Conclusions

In this real-world observational study, we provide long-term evidence on BMD trajectories in patients with rheumatoid arthritis treated with csDMARDs or bDMARDs. BMD remained relatively stable during the first two years of follow-up in both treatment groups.

Beyond two years, modest declines were observed at hip sites, whereas longitudinal analyses suggested a less pronounced decrease in lumbar spine and total hip BMD trajectories among patients receiving bDMARDs compared with those treated with csDMARDs.

These findings underscore the importance of ongoing BMD monitoring and osteoporosis prevention strategies in RA patients, irrespective of disease activity or treatment regimen, to reduce long-term fracture risk and preserve skeletal health.

## Figures and Tables

**Table 1:** Patient characteristics at baseline.

	bDMARD	csDMARD	*p*-Value
Variables	n = 181	n = 131	
Demographic characteristics			
Age, years	57.9 ± 13.4	63.6 ± 12.0	*p* < 0.001
Women, n (%)	154 (85.1)	113 (86.3)	0.77
Postmenopausal women, n (%)	126 (81.8)	100 (88.5)	0.14
Early menopause, n (%)	8 (5.2)	13 (11.5)	0.06
BMI, kg/m^2^	25.9 ± 5.6	26.6 ± 5.0	0.26
Current smoking, n (%)	53 (29.3)	28 (21.4)	0.12
Alcohol usage, n (%)	6 (3.3)	2 (1.5)	0.32
Familial history of femoral neck fracture, n (%)	12 (6.6)	8 (6.1)	0.85
Clinical characteristics			
Disease duration, years	8.1 ±9.4	8.2 ±9.7	0.89
ESR, mm/h	32.1 ± 27.9	34.8 ± 31.3	0.45
DAS28-ESR	4.2 ± 1.5	3.8 ± 1.4	0.02
RF positive, n (%)	142 (80.2)	96 (75.6)	0.33
Anti-CCP positive, n (%)	113 (65.3)	65 (51.2)	0.01
Erosive disease, n (%)	133 (75.1)	74 (57.8)	0.01
Therapeutic characteristics			
bDMARDs:			
TNFα inhibitors, n (%)	119 (65.8)	-	
Anakinra, n (%)	8 (4.4)	-	
Tocilizumab, n (%)	15 (8.3)	-	
Abatacept, n (%)	20 (11.1)	-	
Rituximab, n (%)	19 (10.5)	-	
csDMARDs, n (%)	164 (90.6)	131 (100)	<0.001
Prednisone use, n (%)	145 (80.1)	95 (72.5)	0.12
Prednisone dose, mg/day	6.5 ± 1.9	6.7 ± 2.4	0.58
Duration use, months	81.7 ± 69.8	80.9 ± 74.4	0.94
Calcium and vitamin D3 supplement, n (%)	135 (74.6)	102 (77.9)	0.50
Anti-osteoporosis treatment, n (%)	74(40.9)	43 (33.3)	0.18

bDMARDs: biological disease-modifying antirheumatic drugs; BMI: body mass index; CCP: cyclic citrullinated peptides; csDMARD: conventional synthetic disease-modifying antirheumatic drug; DAS: disease activity score; ESR: erythrocyte sedimentation rate; RF: rheumatoid factor; SD: standard deviation; TNF: tumor necrosis factor. n (%) or mean ± SD.

**Table 2:** Bone characteristics at baseline.

Variables	bDMARD	csDMARD	*p*-Value
Lumbar spine BMD (g/cm^2^)	0.90 ± 0.16	0.92 ± 0.17	0.48
Femoral neck BMD (g/cm^2^)	0.71 ± 0.13	0.69 ± 0.12	0.17
Total hip BMD (g/cm^2^)	0.81 ± 0.15	0.81 ± 0.15	0.79
Lumbar spine t-score	−1.09 ± 1.46	−0.93 ± 1.5	0.36
Femoral neck t-score	−1.39 ± 1.13	−1.56 ± 1.05	0.18
Total hip t-score	−1.12 ± 1.23	−1.12 ± 1.11	0.95

Mean ± SD, bDMARD: biological disease-modifying antirheumatic drug; BMD: bone mineral density; csDMARD: conventional synthetic disease-modifying antirheumatic drug.

**Table 3:** History of fractures and fracture events during follow-up.

		bDMARD (n = 181)	csDMARD (n = 131)	*p*-Value
History of fracture at baseline		21 (11.6%)	16 (12.2%)	0.87
	Vertebral fracture	10 (5.5%)	4 (3.1%)	
	Non-vertebral severe fracture *	4 (2.2%)	4 (3.1%)	
	Minor fracture **	10 (5.5%)	10 (7.6%)	
Fracture events during the follow-up		26 (14.4%)	21 (16.0%)	0.69
	Vertebral fracture	13 (7.2%)	14 (10.7%)	
	Non-vertebral severe fracture *	5 (2.8%)	6 (4.6%)	
	Minor fracture **	9 (5.0%)	6 (4.6%)	

bDMARDs: biological disease-modifying antirheumatic drug; csDMARD: conventional synthetic disease-modifying antirheumatic drug. * Femoral neck, proximal end of humerus, distal end of femur, proximal end of tibia, 3 ribs simultaneous, pelvic fracture excluding fractures of the cervical spine, skull, fingers and toes. ** Fracture of the wrist, distal end of tibia and fibula

**Table 4:** Factors associated with bone mineral density changes over 8 years of follow-up in multivariate linear regression analysis.

	Lumbar Spine		Femoral Neck		Total Hip	
Variables	Adj Beta [CI]	*p*-Value	Adj Beta [CI]	*p*-Value	Adj Beta [CI]	*p*-Value
Age	−0.002 [−0.003; −0.001]	0.001	−0.003 [−0.003; −0.002]	<0.001	−0.002 [−0.002; −0.001]	<0.001
Male sex	0.052 [0.021; 0.083]	0.001	0.069 [0.047; 0.092]	<0.001	0.069 [0.047; 0.092]	<0.001
BMI	0.005 [0.003; 0.007]	<0.001	0.008 [0.007; 0.010]	<0.001	0.008 [0.007; 0.010]	<0.001
Time between each BMD measurement (in months)	−0.0006 [−0.0012; −0.0001]	0.01	−0.0005 [−0.0009; −0.0001]	0.03	−0.0005 [−0.0009; −0.0001]	0.01
Disease duration	0.000 [−0.002; 0.001]	0.51	−0.003 [−0.004; −0.002]	<0.001	−0.003 [−0.004; −0.002]	<0.001
Response to treatment (DAS 28 < 3.2)	0.023 [0.000; 0.047]	0.05	0.007 [−0.010; 0.023]	0.43	0.007 [−0.010; 0.023]	0.44
Treatment with biological therapy	−0.021 [−0.054; 0.011]	0.20	−0.002 [−0.026; 0.021]	0.33	−0.002 [−0.026; 0.021]	0.84
Anti-osteoporosis treatment	−0.053 [−0.077; −0.029]	<0.001	−0.048 [−0.066; −0.030]	<0.001	−0.048 [−0.066; −0.030]	<0.001
Fracture event	-		-		−0.038 [−0.058; −0.018]	<0.001
Corticoids	−0.023 [−0.051; 0.005]	0.11	−0.013 [−0.033; 0.007]	0.09	−0.013 [−0.033; 0.007]	0.22
RA seropositive	−0.046 [−0.076; −0.015]	0.003	−0.009 [−0.031; 0.014]	0.48	−0.009 [−0.031; 0.014]	0.45
Time treatment group	0.001 [0.000; 0.001]	0.004	0.0005 [0.0000; 0.0009]	0.34	0.0005 [0.0000; 0.0009]	0.04

BMD = bone mineral density; BMI = body mass index; CI = confidence interval; DAS: disease activity score; RA: rheumatoid arthritis.

## Data Availability

The data presented in this study are available on request from the corresponding author due to privacy or ethical reasons.

## Abbreviations

The following abbreviations are used in this manuscript:

ACPA	Anti-citrullinated protein antibody
ACR	American college of rheumatology
bDMARD	biological disease-modifying antirheumatic drugs
BMD	Bone mineral density
BMI	Body mass index
CCP	cyclic citrullinated peptides
CI	Confidence interval
csDMARD	conventional synthetic disease-modifying antirheumatic drugs
DAS	Disease activity score
DEXA	Dual-energy X-ray absorptiometry
ESR	Erythrocyte sedimentation rate
EULAR	European League Against Rheumatism
IL-1	Interleukin 1
IL-6	Interleukin 6
Poly.	Polynomial trendline
RA	Rheumatoid arthritis
RANKL	Receptor activator of nuclear factor-kappa B ligand
RF	Rheumatoid factor
SD	Standard deviation
TNFα	Tumor necrosis factor α
